# Acute Kidney Disease in Hospitalized Pediatric Patients With Acute Kidney Injury in China

**DOI:** 10.3389/fped.2022.885055

**Published:** 2022-05-23

**Authors:** Ying-Hao Deng, Ping Yan, Ning-Ya Zhang, Xiao-Qin Luo, Xiu-Fen Wang, Shao-Bin Duan

**Affiliations:** ^1^Department of Nephrology, The Second Xiangya Hospital of Central South University, Hunan Key Laboratory of Kidney Disease and Blood Purification, Changsha, China; ^2^Information Center, The Second Xiangya Hospital of Central South University, Changsha, China

**Keywords:** acute kidney injury, acute kidney disease, pediatric, outcomes, mortality, epidemiology

## Abstract

**Objective:**

The epidemiology and outcomes of acute kidney disease (AKD) after acute kidney injury (AKI) in hospitalized children are poorly described. The aim of this study is to investigate the prevalence, predictive factors, and clinical outcomes of AKD in hospitalized children with AKI.

**Methods:**

Children (1 month–18 years) with AKI during hospitalization in the Second Xiangya Hospital from January 2015 to December 2020 were identified. AKD was defined based on the consensus report of the Acute Disease Quality Initiative 16 workgroup. The endpoints include adverse outcomes in 30 and 90 days. Multivariable logistic regression analyses were used to estimate the odds ratio of 30- and 90-day adverse outcomes associated with AKD and identify the risk factors of AKD.

**Results:**

AKD was developed in 42.3% (419/990) of the study patients, with 186 in AKD stage 1, 107 in AKD stage 2, and 126 in AKD stage 3. Pediatric patients with AKD stages 2–3 had significantly higher rates of developing 30- and 90-day adverse outcomes than those with AKD stage 0 and 1. The adjusted odds ratio of AKD stage 2–3 was 12.18 (95% confidence interval (CI), 7.38 - 20.09) for 30-day adverse outcomes and decreased to 2.49 (95% CI, 1.26 - 4.91) for 90-day adverse outcomes. AKI stages 2 and 3, as well as glomerulonephritis, were the only predictive factors for AKD stage 2–3.

**Conclusion:**

AKD is frequent among hospitalized pediatric AKI patients. AKD stage 2–3 represents a high-risk subpopulation among pediatric AKI survivors and is independently associated with 30- and 90-day adverse outcomes. Awareness of the potential risks associated with AKD stage 2–3 and its risk factors may help improve outcomes through careful monitoring and timely intervention.

## Introduction

Acute kidney injury (AKI) is a public health problem and is common in children. AKI could occur in 27% of hospitalized children admitted to intensive care units (ICUs), with 12% of children developing AKI stage 2 and 3 (1). And the overall incidences of community-acquired AKI and hospitalized-acquired AKI were estimated to be 7 and 13%, respectively (2). AKI was independently associated with death, progression of chronic kidney disease (CKD), as well as longer ICU and hospital stay (3). However, few methods have been shown to effectively prevent or revere AKI in clinical practice (4, 5). Therefore, understanding the clinical epidemiology and the pattern of renal progression/recovery after an episode of AKI is important.

Acute kidney disease (AKD) proposed by the Acute Disease Quality Initiative (ADQI) 16 Workgroup describes acute or subacute damage and/or loss of kidney function for 7 to 90 days after exposure to an AKI initiating event (6). AKD represents the course of disease among AKI patients in whom the renal pathophysiologic process is still ongoing and seems to represent the intermediate stage between AKI and CKD. The epidemiology and outcomes of AKD have been investigated extensively in adult patients and AKD was found to be independently associated with increased risks of CKD, dialysis, and mortality (7–10). However, the epidemiology and the impact of AKD on adverse outcomes in pediatric patients with AKI have been poorly described.

To address this issue, we conducted an epidemiological study of AKD in hospitalized Children with AKI in China. We aimed to assess the prevalence and predictive factors of AKD, and the impact of AKD on 30- and 90-day adverse outcomes in hospitalized pediatric patients with AKI.

## Materials and Methods

### Study Population

**FIGURE 1:**
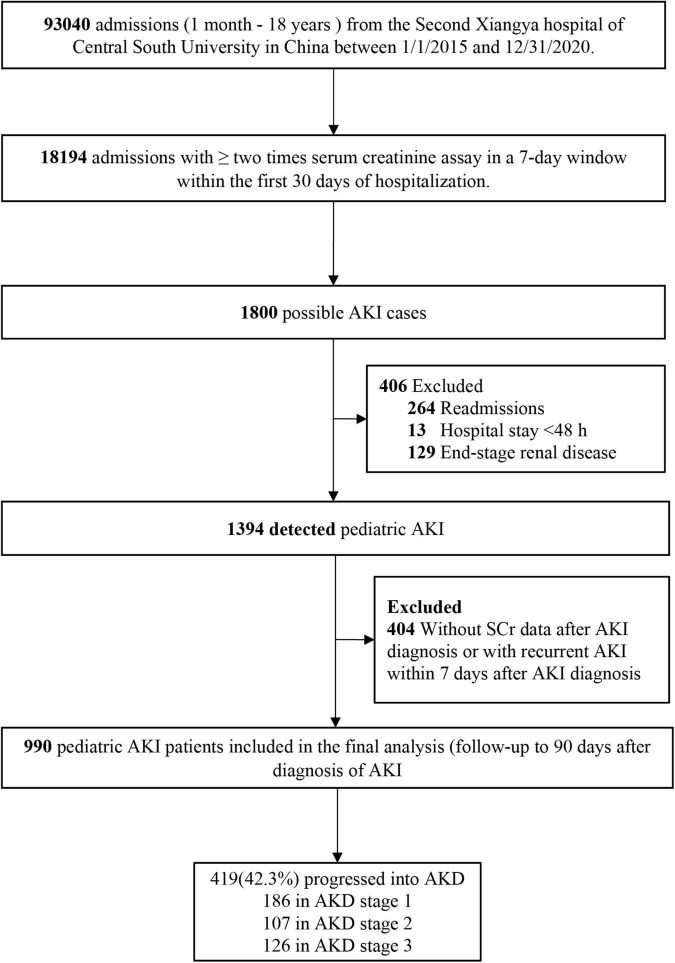
Study flowchart. AKI, acute kidney injury; AKD, acute kidney disease.

This retrospective cohort study identified pediatric AKI patients admitted to the Second Xiangya Hospital of Central South University in China from January 1, 2015, to December 31, 2020. Patients aged between 1 month and 18 years were included. Possible pediatric patients with AKI were screened based on the change in serum creatinine (SCr) through Laboratory Information System and Hospital Information System. The details of patient selection are shown in Figure 1. AKI was defined based on the SCr criteria of the 2012 Kidney Disease: Improving Global Outcomes (KDIGO) Clinical Practice Guideline as an increase in SCr by ≥26.5 μmol/L within 48 h or to ≥1.5 times baseline within 7 days (11). Baseline SCr was defined as the lowest SCr in the 7 days prior to AKI diagnosis, or the minimum inpatient SCr value for patients who met the criteria of community-acquired AKI (2). Recurrent AKI within 7 days was defined as the case when SCr dropped to ≤baseline SCr and then the second episode of AKI occurred within 7 days after AKI diagnosis. Only the first hospitalization was included if pediatric patients had multiple hospitalizations. The study was approved by the Medical Ethics Committee of the Second Xiangya Hospital of Central South University and informed consent was waived. This project has been registered in the Chinese Clinical Trial Registry (2013S061).

### Determination of Acute Kidney Disease

Acute kidney disease was defined as a condition in which AKI stage 1 or greater persists between 6 and 90 days after an AKI initiating event according to the consensus report of the ADQI 16 Workgroup (6). KDIGO AKI staging categories were mapped to the staging of AKD (11). We identified AKD and AKD stage on AKD diagnosis (hereafter referred to as AKD stage) based on the SCr level on the 7th day or the day nearest to the 7th day after the diagnosis of AKI. Patients without AKD were classified as AKD stage 0.

### Data Collection

Patients’ data, including demographics, comorbidities, laboratory test results, medication, and hospital procedures, were extracted from the hospital electronic medical record system and laboratory information system. AKI stage was defined according to the KDIGO criteria and determined using the highest SCr value during the first 7 days after the diagnosis of AKI. Community-acquired AKI was determined when the increase in SCr on the first day met the KDIGO criteria, or the SCr value on admission was ≥1.5 times the baseline SCr value. Patients who did not meet the criteria for community-acquired AKI were categorized as hospital-acquired AKI. Comorbidities were identified according to the diagnosis codes (International Classification of Diseases, 10th Edition) on admission and at discharge. The data of laboratory tests, nephrotoxic drugs as well as clinical interventions were collected within 7 days after AKI diagnosis. Nephrotoxic drugs included non-steroidal anti-inflammatory drugs, proton pump inhibitors, antimycotics, contrast media, aminoglycoside antibiotics, and chemotherapeutic drugs (2). The information on surgeries was extracted prior to the diagnosis of AKD. If multiple values were available for the same laboratory parameter, the one closest to the time of AKI diagnosis was used. Clinical interventions included the use of loop diuretics (furosemide, torasemide, bumetanide, and ethacrynic acid), mechanical ventilation, and renal replacement therapy (RRT).

### Outcomes

The primary outcomes were Major Adverse Kidney Events within 30 days (MAKE30) and 90-day adverse outcomes. MAKE30 was defined as any of or a composite of mortality and/or new receipt of RRT and/or persistent renal dysfunction within 30 days after AKI diagnosis (12, 13). Persistent renal dysfunction was defined as the SCr value ≥2 times the baseline value. The 90-day adverse outcomes was defined as any of or a composite of mortality and chronic dialysis within 90 days after the diagnosis of AKI. Survival status after hospital discharge was obtained from the Chinese Center for Disease Control and Prevention cause–of–death reporting system. Chronic dialysis was determined by reviewing patients’ medical records, making phone calls, and referring to the Chinese National Renal Data System. The secondary outcome was the length of hospital stay after AKI diagnosis, defined as the number of days between AKI diagnosis and hospital discharge.

### Statistical Analysis

Quantitative variables were presented as medians (interquartile range) and categorical data as frequencies (percentages). Pediatric patients’ baseline characteristics and outcomes were compared using Mann–Whitney U test for continuous variables and chi-square test for categorical variables. The Kaplan–Meier method was used to compare the 90-day survival among AKD stages and the differences were estimated with log - rank test. Multivariable logistic regression analysis was used to identify the independent risk factors for MAKE30 and 90-day adverse outcomes. Multivariable Cox regression analysis was used to identify the independent risk factors for mortality in 30 and 90 days. Variables that were considered to be relevant to the outcomes clinically or with *p* < 0.05 in univariate analysis were included in the multivariable analysis.

## Results

### Incidence of Acute Kidney Disease

A total of 990 pediatric patients with AKI were identified in our study. AKD occurred in 42.3% (419/990) of children with AKI, with 186 in AKD stage 1, 107 in AKD stage 2, and 126 in AKD stage 3. The baseline characteristics of the study population stratified by AKD stages are presented in Table 1. A total of 127 AKD children (30.3%) occurred in AKI stage 1, 130 (31.0%) in AKI stage 2 and 162 (38.7%) in AKI stage 3. Compared with those with AKD stage 0 and 1, pediatric patients with AKD stage 2–3 were older and more likely to develop community- acquired AKI. Significantly larger percentages of children with glomerular nephritis, nephrotic syndrome, CKD, cardiac surgery, heart failure as well as cardiac arrest were also observed in AKD stage 2–3.

**TABLE 1:** Baseline characteristics of the study population.

Variables	AKD stage 0	AKD stage 1	AKD stage 2–3	*P*-value
	*N* = 571	*N* = 186	*N* = 233	
Age	5.00 [0.00;12.0]	6.00 [1.00;12.0]	9.00 [2.00;14.0]	<0.001
**Age group**				0.001
Infancy, 1 mo −1 yr	188 (32.9%)	49 (26.3%)	55 (23.6%)	
Childhood, 2 −10 yr	216 (37.8%)	76 (40.9%)	75 (32.2%)	
Adolescence, 11–18 yr	167 (29.2%)	61 (32.8%)	103 (44.2%)	
Male	323 (56.6%)	104 (55.9%)	148 (63.5%)	0.155
**AKI type**				0.001
Community–acquired AKI	166 (29.1%)	69 (37.1%)	99 (42.5%)	
Hospital–acquired AKI	405 (70.9%)	117 (62.9%)	134 (57.5%)	
**AKI stage**				<0.001
Stage 1	395 (69.2%)	108 (58.1%)	19 (8.15%)	
Stage 2	126 (22.1%)	61 (32.8%)	69 (29.6%)	
Stage 3	50 (8.76%)	17 (9.14%)	145 (62.2%)	
**Comorbidities**				
Sepsis	96 (16.8%)	34 (18.3%)	34 (14.6%)	0.584
Glomerulonephritis	13 (2.28%)	7 (3.76%)	28 (12.0%)	<0.001
Nephrotic syndrome	48 (8.41%)	23 (12.4%)	59 (25.3%)	<0.001
Chronic kidney disease	4 (0.70%)	2 (1.08%)	7 (3.00%)	0.036
Urinary tract obstruction/malformation	11 (1.93%)	6 (3.23%)	2 (0.86%)	0.220
Non-cardiac surgery	38 (6.65%)	11 (5.91%)	5 (2.15%)	0.037
Cardiac surgery	175 (30.6%)	32 (17.2%)	57 (24.5%)	0.001
Heart failure	50 (8.76%)	12 (6.45%)	33 (14.2%)	0.017
Inherited metabolic disease	9 (1.58%)	2 (1.08%)	4 (1.72%)	0.938
Cardiac arrest	3 (0.53%)	0 (0.00%)	6 (2.58%)	0.013
Trauma/burn	11 (1.93%)	6 (3.23%)	5 (2.15%)	0.531
Shock	26 (4.55%)	9 (4.84%)	19 (8.15%)	0.115
Respiratory failure	56 (9.81%)	18 (9.68%)	30 (12.9%)	0.402
Acute diarrhea/dehydration	18 (3.15%)	14 (7.53%)	12 (5.15%)	0.035
Nephrotoxic medicine	293 (51.3%)	94 (50.5%)	103 (44.2%)	0.179
**Laboratory data**				
Anemia	148 (25.9%)	48 (25.8%)	62 (26.6%)	0.976
Thrombocytopenia	119 (20.8%)	30 (16.1%)	57 (24.5%)	0.113
Proteinuria	90 (18.7%)	40 (25.5%)	95 (48.7%)	<0.001
Hypoalbuminemia	133 (23.3%)	48 (25.9%)	114 (48.9%)	<0.001
Hyperbilirubinemia	74 (13.0%)	14 (7.61%)	30 (12.9%)	0.130
Hyperkalemia	22 (3.97%)	8 (4.42%)	16 (6.96%)	0.197
Use of diuretics	320 (56.0%)	64 (34.4%)	159 (68.2%)	<0.001
Mechanical ventilation	143 (25.0%)	31 (16.7%)	65 (27.9%)	0.021

*AKD, acute kidney disease; AKI, acute kidney injury.*

The comparison of baseline characteristics between patients included in our study and those excluded due to inadequate SCr data or recurrent AKI is provided in Supplementary Table 1. Compared with the patients included in our study, the excluded patients were younger, more likely to be hospital-acquired AKI and AKI stage 1, and had lower proportions in some of the comorbidities.

### Outcomes of Acute Kidney Disease

**FIGURE 2:**
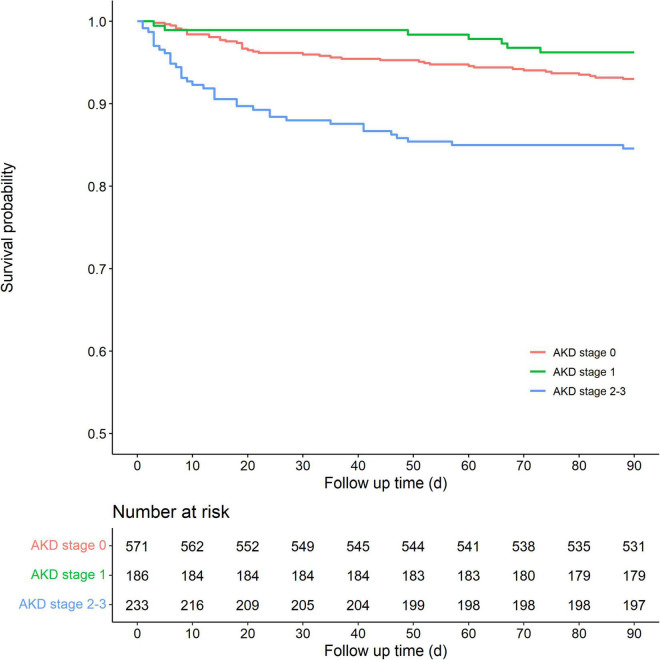
Kaplan–Meier survival curves for 90 days mortality among AKD stages. The overall *p-*value was <0.001 among the three groups. The *p-*value for pairwise comparison was 0.112 between AKD stage 0 and AKD stage 1, <0.001 between AKD stage 0 and AKD stage 2–3 and between AKD stage 1 and AKD stage 2–3. AKD, acute kidney disease.

Table 2 shows the outcomes of patients stratified by AKD stage. The length of hospital stay after AKI diagnosis was similar among pediatric patients with different AKD stage. Pediatric patients with AKD stage 0 and AKD stage 1 showed similar incidences in the composite endpoints of MAKE30 (11.9% vs. 12.4%) and 90-day adverse outcomes (7.0% *vs.* 4.3%). However, pediatric patients with AKD stage 2–3 had significantly higher incidences of developing all adverse outcomes in 30 and 90 days with the incidence of MAKE30 and 90-day adverse outcomes being 67.8 and 20.2%, respectively. Notably, the incidences of mortality in 30 and 90 days were as high as 12.0 and 15.5% in the AKD stage 2–3 pediatric patients, respectively. Kaplan–Meier curves showed that pediatric patients with AKD stage 2–3 had a significantly lower 90-day survival rate than those with AKD stage 0 (pairwise comparison: *p* < 0.001) and AKD stage 1 (pairwise comparison: *p* < 0.001). However, there was no statistical difference in the survival between AKD stage 0 and 1 (pairwise comparison: *p* = 0.112) (Figure 2).

**TABLE 2:** Outcomes of the study population stratified by AKD stage.

Outcomes	AKD stage 0	AKD stage 1	AKD stage 2–3	*p-*Value
	*N* = 571	*N* = 186	*N* = 233	
Length of hospital stay after AKI diagnosis	20.0 [11.0;30.5]	19.0 [11.0;36.5]	21.0 [12.0;36.0]	0.339
**Major adverse kidney events within 30 days**				
PRD	30 (5.3%)	10 (5.4%)	112 (48.1%)	<0.001
Receipt of new RRT	29 (5.1%)	11 (5.9%)	81 (34.8%)	<0.001
Mortality	22 (3.9%)	2 (1.1%)	28 (12.0%)	<0.001
Total	68 (11.9%)	23 (12.4%)	158 (67.8%)	<0.001
**90-day adverse outcomes**				
Mortality	40 (7.0%)	7 (3.8%)	36 (15.5%)	<0.001
Chronic dialysis	0 (0.0%)	1 (0.5%)	11 (4.7%)	<0.001
Total	40 (7.0%)	8 (4.3%)	47 (20.2%)	<0.001

*AKD, acute kidney disease; PRD, persistent renal dysfunction; RRT: renal replacement therapy.*

Association between AKD stage and 30- and 90-day adverse outcomes are shown in Tables 3 and 4. After adjustment for other confounding variables, the odds ratio for AKD stage 2–3 was as high as 12.18 in the multivariable logistic regression analysis for developing MAKE30, and the risk of developing 90-day adverse outcomes was still 2.49 times higher than AKD stage 0. Other predictors for MAKE30 included AKI stage 3, shock, respiratory failure, hypoalbuminemia, hyperbilirubinemia as well as hyperkalemia. Age, CKD, shock, respiratory failure, thrombocytopenia, hyperbilirubinemia, and mechanical ventilation were risk factors for 90-day adverse outcomes. Association between AKD stage and 30- and 90-day mortality is shown in Supplementary Tables 2, 3. AKD stage 2–3 was associated with a 3-fold increased risk in developing 30-day mortality and still carried an 1.96-fold increased risk in developing 90-day mortality.

**TABLE 3:** Multivariable logistic analysis for predictors of Major Adverse Kidney Events within 30 days.

Variables	odds ratio	95% CI	*p-*Value
**AKD stage**			
0	Reference	–	–
1	1.06	0.61–1.86	0.83
2–3	12.18	7.38–20.09	<0.001
Age	0.99	0.96–1.03	0.68
Hospital-acquired AKI	0.89	0.60–1.32	0.56
**AKI stage**			
1	Reference	–	–
2	1.55	0.97–2.46	0.07
3	1.93	1.11–3.35	0.019
Glomerulonephritis	1.21	0.49–3.04	0.68
Nephrotic syndrome	0.56	0.25–1.26	0.16
Heart failure	1.14	0.61–2.12	0.68
Shock	2.24	1.02–4.92	0.044
Respiratory failure	2.38	1.31–4.30	0.004
Anemia	1.54	0.95–2.50	0.08
Thrombocytopenia	1.03	0.63–1.71	0.90
Proteinuria	1.55	0.88–2.73	0.13
Hypoalbuminemia	1.77	1.07–2.91	0.025
Hyperbilirubinemia	1.86	1.05–3.31	0.034
Hyperkalemia	2.40	1.06–5.40	0.035
Use of diuretics	1.09	0.69–1.71	0.72
Mechanical ventilation	2.02	1.23–3.33	0.06

*CI, confidence interval; AKD, acute kidney disease; AKI, acute kidney injury.*

**TABLE 4:** Multivariable logistic analysis for predictors of 90-day adverse outcomes.

Variables	Odds ratio	95% CI	*p-*Value
**AKD stage**			
0	Reference	–	–
1	0.53	0.22–1.26	0.15
2–3	2.49	1.26–4.91	0.009
Age	1.07	1.02–1.11	0.003
**AKI stage**			
1	Reference	–	–
2	1.16	0.60–2.24	0.65
3	1.02	0.46–2.25	0.97
Chronic kidney disease	8.97	2.48–32.51	<0.001
Heart failure	0.82	0.37–1.81	0.63
Shock	3.07	1.35–6.99	0.007
Respiratory failure	3.34	1.69–6.59	<0.001
Anemia	1.25	0.69–2.25	0.47
Thrombocytopenia	1.91	1.05–3.45	0.033
Proteinuria	1.38	0.72–2.64	0.33
Hypoalbuminemia	1.46	0.84–2.53	0.18
Hyperbilirubinemia	2.18	1.08–4.41	0.031
Use of diuretics	0.79	0.42–1.50	0.47
Mechanical ventilation	3.38	1.78–6.41	<0.001

*CI, confidence interval; AKD, acute kidney disease; AKI, acute kidney injury.*

### Prediction of Acute Kidney Disease Stage 2–3

Multivariable logistic regression analysis showed that the AKI stage was the major predictor for AKD stage 2–3 and predicted it in a graded manner with the odds ratio of AKI stage 2 and AKI stage 3 being 8.88 (95% confidence interval [95%CI], 5.15–15.34) and 45.71 (95% CI, 26.05–80.22), respectively (Table 5). Glomerulonephritis was also found to be independently associated with the development of AKD stage 2–3 with the odds ratio being 2.70 (95% CI, 1.10–6.65).

**TABLE 5:** Multivariable logistic analysis for predictors of AKD stage 2–3.

Variables	Odds Ratio	95% CI	*p-*Value
Age	1.01	0.98–1.05	0.41
Hospital-acquired AKI	0.89	0.59–1.33	0.56
**AKI stage**			
1	1.00	–	–
2	8.88	5.15–15.34	<0.001
3	45.71	26.05–80.22	<0.001
Glomerulonephritis	2.70	1.10–6.65	0.030
Nephrotic syndrome	1.62	0.75–3.49	0.22
Chronic kidney disease	1.55	0.32–7.57	0.59
Non-cardiac surgery	0.63	0.21–1.85	0.40
Heart failure	1.00	0.53–1.89	0.99
Cardiac arrest	4.39	0.66–29.19	0.13
Proteinuria	1.27	0.71–2.28	0.41
Hypoalbuminemia	1.26	0.75–2.11	0.38
Use of diuretics	0.97	0.64–1.47	0.88

*AKD, acute kidney disease; CI, confidence interval; AKI, acute kidney injury.*

### Sensitivity Analysis

To test whether the association between AKD and adverse outcomes in 30 and 90 days would be affected due to the exclusion of pediatric patients with inadequate SCr data or recurrent AKI, we included the excluded patients as AKD stage 0 in our data set as they were mostly in AKI stage 1 and in less severe conditions. Logistic regression analysis was performed and the results were presented in Supplementary Tables 4, 5. AKD stage 2–3 is still associated with an increased risk of MAKE30 and 90-day adverse outcomes with the odds ratio being 4.30 (95%CI, 2.79–6.64) and 1.89 (95%CI, 1.02–3.51), respectively. The association between AKD and adverse outcomes remained stable in our study.

## Discussion

In our study, we mainly provided three results in relation to AKD in hospitalized pediatric patients with AKI: (1) the prevalence, (2) the predictive factors, (3) the impact of AKD on 30- and 90-day adverse outcomes. We found that 42.3% of hospitalized pediatric patients with AKI developed AKD. AKD stage 2–3 was independently associated with increased risks of MAKE30 and 90-day adverse outcomes, and AKI stage and glomerulonephritis were its predictors.

Studies in relation to AKD in pediatric patients have been scarce. Patel et al. investigated the epidemiology of AKD in children after transplant, where AKD was defined as impaired kidney function lasting for <90 days with or without an AKI event and found that AKD occurred in 13% of patients and was independently associated with new-onset CKD (14). LoBasso et al. explored the kidney recovery patterns of AKI and the relationship with hospital outcomes in children undergoing cardiopulmonary bypass (15). The study found that AKD developed in 3.1% of patients with AKI and was the subgroup that had the highest mortality. Compared with those studies, our research conducted an original and innovative observation of AKD after AKI in pediatric patients. We included all hospitalized children with AKI and found that AKD was common as 42.3% of children progressed into AKD. In addition, AKD stage 2–3 was found to be associated with increased risks of MAKE30 and 90-day adverse outcomes. The inclusion of all eligible patients from all departments in our research made the study population more representative of the real AKD group. The differences in the incidence of AKD reported in previous studies and our study could be explained by the different definitions of AKD as well as the study population.

Our findings have important clinical implications. First, we depicted the clinical course of AKI – AKD – MAKE30 – 90-day adverse outcomes, aiming to provide information on the renal function progression after AKI in pediatric patients. We found that a further progression of renal injury after AKI could add the risk of 30- and 90-day adverse outcomes, which supported the results of our previous study of AKD in hospitalized adult patients with AKI (8). AKI stage 2–3, which was generally considered to be associated with increased risks of CKD, kidney failure, and mortality (1, 16–18), was no longer a predictor for adverse outcomes in our multivariable logistic regression analysis. However, AKD stage 2–3 became the strongest predictor of MAKE30 with odds ratio being 12.18 (95%CI, 7.38–20.09) and still carried a 2.49–fold increased risk of adverse 90 days outcomes. The findings confirmed that AKD served as an intermediate step between AKI and CKD (6). Our results were also consistent with previous studies in adult patients, which indicated that patients without recovery after AKI were at increased risks for CKD and death both in the short term and long term (19–21).

Second, as patients with AKD stage 2–3 represented a subpopulation with a higher risk for adverse outcomes among pediatric AKI survivors, it’s essential to raise awareness of AKD stage 2–3 and early identify these patients. Strengthening training and education about the knowledge of acute and subacute kidney disease to all healthcare workers is of great importance in providing timely interventions and improving outcomes. Timely treatment of the causes of AKI, avoidance of nephrotoxic medicine and radiocontrast procedures, ensuring volume status and perfusion pressure, and close monitoring of renal function and urine output in pediatric patients with AKD stage 2–3 are suggested.

Third, AKI stage 2 and 3 and glomerulonephritis were independent predictors of AKD stage 2–3 in pediatric patients. Therefore, patients with these risk factors should be given careful care and close follow-up.

Finally, as AKD may represent the important transition period between AKI and CKD, research in elucidating the pathophysiological changes and mechanism of AKD can help increase understanding of AKD and find out possible intervening measures to stop renal progression and facilitate recovery.

Our study has several limitations. First, because the information on urinary output was largely unavailable in hospitalized pediatric patients, we did not use the criterion of urine output to identify AKI, which could miss some possible AKI cases. Second, the study patients were only enrolled from a single center which could reduce the generalizability of the study population. Third, children without enough SCr data after AKI diagnosis or with recurrent AKI were excluded from our study. These pediatric patients were in less severe conditions than patients included in our study, which could result in the overestimation of the incidence of AKD. Fourth, the potentially unmeasured variables in this retrospective study might confound the association between AKD and adverse outcomes. Finally, cause-specific mortality was not identified in our research. Renal-associated death could better explain the association between AKD and renal prognosis. Large multicenter studies, especially prospective studies are needed to validate our findings and resolve the limitations.

## Conclusion

Acute kidney disease is commonly seen in hospitalized pediatric AKI patients. AKD stage 2–3 is independently associated with increased risks of 30- and 90-day adverse outcomes, which are mainly predicted by the AKI stage. Awareness of potential risks associated with AKD stage 2–3 and its risk factors may help improve outcomes through careful monitoring and timely intervention.

## Data Availability Statement

The raw data supporting the conclusions of this article will be made available by the authors, without undue reservation.

## Ethics Statement

The studies involving human participants were reviewed and approved by the Medical Ethics Committee of the Second Xiangya Hospital of Central South University. Written informed consent from the participants’ legal guardian/next of kin was not required to participate in this study in accordance with the national legislation and the institutional requirements.

## Author Contributions

S-BD contributed to the study design and supervision. Y-HD, PY, X-QL, N-YZ, and X-FW contributed to the data acquisition, data analysis, data interpretation, and statistical analysis. Y-HD and PY contributed to the writing and revision of the manuscript. X-QL, N-YZ, X-FW, and S-BD contributed to the revision of the manuscript. All authors approved the final version of the manuscript and agreed to be accountable for all aspects of the work.

## Conflict of Interest

The authors declare that the research was conducted in the absence of any commercial or financial relationships that could be construed as a potential conflict of interest.

## Publisher’s Note

All claims expressed in this article are solely those of the authors and do not necessarily represent those of their affiliated organizations, or those of the publisher, the editors and the reviewers. Any product that may be evaluated in this article, or claim that may be made by its manufacturer, is not guaranteed or endorsed by the publisher.
