# Supplementary material for: Acute Kidney Disease in Hospitalized Pediatric Patients With Acute Kidney Injury in China
Source: Front Pediatr. 2022 May 23;10:885055. doi: 10.3389/fped.2022.885055 (PMC9168069; doi:10.3389/fped.2022.885055)
Supplement: Supplementary file 1 [file Data_Sheet_1.pdf]

Supplementary Table 1. Comparison of baseline characteristics between patients included in the study and those excluded due to inadequate SCr data or recurrent AKI

Variables	Included<br>N=990	Excluded<br>N=404	P value
Age	6.0 [1.0-12.0]	1.0 [0.0-6.0]	<0.001
**Age group**			<0.001
infancy, 1 mo -1yr	292 (29.5%)	212 (52.5%)	
Childhood, 2-10 yr	367 (37.1%)	135 (33.4%)	
Adolescence, 11-18yr	331 (33.4%)	57 (14.1%)	
Male	575 (58.1%)	242 (59.9%)	0.531
**AKI type**			<0.001
Community-acquired AKI	334 (33.7%)	9 (2.2%)	
Hospital-acquired AKI	656 (66.3%)	395 (97.8%)	
**AKI stage**			<0.001
Stage 1	522 (52.7%)	322 (79.7%)	
Stage 2	256 (25.9%)	63 (15.6%)	
Stage 3	212 (21.4%)	19 (4.7%)	
**Comorbidities**			
Sepsis	164 (16.6%)	17 (4.2%)	<0.001
Glomerulonephritis	48 (4.8%)	9 (2.2%)	0.025
Nephrotic syndrome	130 (13.1%)	26 (6.4%)	<0.001
Chronic kidney disease	13 (1.3%)	2 (0.5%)	0.255
Urinary tract obstruction/ malformation	19 (1.9%)	6 (1.5%)	0.580
Non-cardiac surgery	54 (5.5%)	18 (4.5%)	0.444
Cardiac surgery	264 (26.7%)	187 (46.3%)	<0.001
Heart failure	95 (9.6%)	19 (4.7%)	0.002
Inherited metabolic disease	15 (1.5%)	9 (2.2%)	0.353
Cardiac arrest	9 (0.9%)	3 (0.7%)	0.760
Trauma/burn	22 (2.2%)	5 (1.2%)	0.226
Shock	54 (5.5%)	7 (1.7%)	0.002
Respiratory failure	104 (10.5%)	16 (4.0%)	<0.001

Acute diarrhea/ dehydration	44 (4.4%)	12 (3.0%)	0.204
Nephrotoxic medicine	490 (49.5%)	183 (45.3%)	0.155
**Laboratory data**			
Anemia	258 (26.1%)	55 (13.6%)	<0.001
Thrombocytopenia	206 (20.8%)	33 (8.2%)	<0.001
Proteinuria	225 (27.0%)	36 (11.4%)	<0.001
Hypoalbuminemia	295 (29.9%)	37 (9.2%)	<0.001
Hyperbilirubinemia	118 (12.0%)	38 (9.5%)	0.183
Hyperkalemia	46 (4.8%)	43 (10.8%)	<0.001
Use of diuretics	543 (54.8%)	193 (47.8%)	0.016
Mechanical ventilation	239 (24.1%)	35 (8.7%)	<0.001

AKI, acute kidney injury

Supplementary Table 2. Multivariable Cox regression analysis for predictors of 30-day mortality

Variables	Hazard Ratio	95% CI	P value
**AKD stage**			
0	Reference	-	-
1	0.43	0.09-2.01	0.280
2-3	3.00	1.11-8.08	0.030
Age	1.08	1.01-1.14	0.015
Hospital-acquired AKI	6.36	1.89-21.39	0.003
**AKI stage**			
1	Reference	-	-
2	0.96	0.37-2.53	0.941
3	0.77	0.24-2.51	0.663
Nephrotic syndrome	0.32	0.06-1.75	0.188
Heart failure	1.67	0.69-4.00	0.253
Cardiac arrest	2.04	0.46-9.14	0.349
Trauma/burn	2.16	0.41-11.28	0.362
Shock	1.64	0.64-4.20	0.303
Respiratory failure	4.29	1.75-10.52	0.001
Nephrotoxic medicine	0.57	0.22-1.46	0.240
Anemia	2.83	1.34-5.98	0.006
Thrombocytopenia	0.72	0.33-1.55	0.397
Proteinuria	2.71	1.23-5.94	0.013
Hypoalbuminemia	1.27	0.64-2.55	0.495
Hyperbilirubinemia	2.20	0.93-5.22	0.074
Hyperkalemia	4.45	1.48-13.42	0.008
Use of diuretics	1.14	0.40-3.27	0.809
Mechanical ventilation	5.15	1.93-13.78	0.001

CI, confidence interval; AKD, acute kidney disease; AKI, acute kidney injury.

Supplementary Table 3. Multivariable Cox regression analysis for predictors of 90-day mortality

Variables	Hazard Ratio	95% CI	P value
**AKD stage**			
0	reference		
1	0.56	0.25-1.30	0.179
2-3	1.96	1.08-3.59	0.028
Age	1.05	1.01-1.08	0.017
**AKI stage**			
1	reference		
2	1.32	0.73-2.38	0.357
3	0.87	0.41-1.85	0.711
Nephrotic syndrome	0.50	0.16-1.56	0.233
Heart failure	1.23	0.67-2.27	0.505
Cardiac arrest	0.89	0.25-3.09	0.849
Shock	2.01	1.03-3.94	0.041
Respiratory failure	2.43	1.34-4.42	0.004
Nephrotoxic medicine	1.28	0.80-2.05	0.304
Use of diuretics	0.95	0.51-1.76	0.871
Mechanical ventilation	3.36	1.83-6.20	<0.001
Thrombocytopenia	1.41	0.83-2.42	0.207
Anemia	1.48	0.88-2.47	0.139
Hypoalbuminemia	1.42	0.86-2.34	0.169
Hyperbilirubinemia	1.65	0.92-2.95	0.094

CI, confidence interval; AKD, acute kidney disease; AKI, acute kidney injury.

Supplementary Table 4. Multivariable logistic analysis for predictors of Major Adverse Kidney Events within 30 days in 1394 pediatric patients with AKI

Variables	Odds Ratio	95% CI	P value
**AKD stage**			
0	Reference	-	-
1	0.50	0.29-0.85	0.011
2-3	4.30	2.79-6.64	<0.001
Age	0.99	0.96-1.02	0.475
Hospital-acquired AKI	1.72	1.12-2.63	0.013
**AKI stage**			
1	Reference	-	-
2	7.85	5.37-11.48	<0.001
3	8.30	5.16-13.35	<0.001
Glomerulonephritis	1.19	0.53-2.71	0.673
Nephrotic syndrome	0.47	0.22-0.99	0.048
Heart failure	1.20	0.70-2.07	0.502
Cardiac arrest	1.64	0.34-7.98	0.544
Shock	1.86	0.88-3.91	0.102
Respiratory failure	2.46	1.04-4.32	0.002
Nephrotoxic drugs	0.67	0.46-0.98	0.038
Proteinuria	1.42	0.85-2.38	0.179
Use of diuretics	0.87	0.60-1.27	0.473
Mechanical ventilation	1.20	0.79-1.84	0.398
Anemia	1.18	0.77-1.82	0.447
Thrombocytopenia	1.20	0.77-1.87	0.415
Hypoalbuminemia	1.68	1.04-2.71	0.033
Hyperbilirubinemia	1.94	1.21-3.12	0.006
Hyperkalemia	1.93	1.07-3.51	0.030

CI, confidence interval; AKD, acute kidney disease; AKI, acute kidney injury.

Supplementary Table 5. Multivariable logistic analysis for predictors of 90-day adverse outcomes in 1394 pediatric patients with AKI

Variables	Odds Ratio	95% CI	P value
**AKD stage**			
0	Reference	-	-
1	0.46	0.20-1.08	0.074
2-3	1.89	1.02-3.51	0.042
Age	1.09	1.05-1.13	<0.001
**AKI stage**			
1	Reference	-	-
2	1.75	0.98-3.13	0.060
3	1.93	0.95-3.92	0.068
Chronic kidney disease	12.35	3.78-40.35	<0.001
Sepsis	0.67	0.32-1.38	0.275
Glomerulonephritis	0.56	0.21-1.50	0.249
Cardiac arrest	1.16	0.24-5.73	0.854
Heart failure	0.89	0.43-1.84	0.752
Shock	4.92	2.17-11.18	<0.001
Respiratory failure	2.87	1.54-5.35	0.001
Anemia	1.56	0.94-2.58	0.084
Proteinuria	1.13	0.65-1.99	0.665
Use of diuretics	0.94	0.54-1.61	0.810
Mechanical ventilation	3.51	2.00-6.13	<0.001
Hyperkalemia	2.76	1.26-6.04	0.011

CI, confidence interval; AKD, acute kidney disease; AKI, acute kidney injury.
